# Calcium Imaging of Neuronal Activity in *Drosophila* Can Identify Anticonvulsive Compounds

**DOI:** 10.1371/journal.pone.0148461

**Published:** 2016-02-10

**Authors:** Anne K. Streit, Yuen Ngan Fan, Laura Masullo, Richard A. Baines

**Affiliations:** Faculty of Life Sciences, University of Manchester, Oxford Road, Manchester, M13 9PT, United Kingdom; EPFL, SWITZERLAND

## Abstract

Although there are now a number of antiepileptic drugs (AEDs) available, approximately one-third of epilepsy patients respond poorly to drug intervention. The reasons for this are complex, but are probably reflective of the increasing number of identified mutations that predispose individuals to this disease. Thus, there is a clear requirement for the development of novel treatments to address this unmet clinical need. The existence of gene mutations that mimic a seizure-like behaviour in the fruit fly, *Drosophila melanogaster*, offers the possibility to exploit the powerful genetics of this insect to identify novel cellular targets to facilitate design of more effective AEDs. In this study we use neuronal expression of GCaMP, a potent calcium reporter, to image neuronal activity using a non-invasive and rapid method. Expression in motoneurons in the isolated CNS of third instar larvae shows waves of calcium-activity that pass between segments of the ventral nerve cord. Time between calcium peaks, in the same neurons, between adjacent segments usually show a temporal separation of greater than 200 ms. Exposure to proconvulsants (picrotoxin or 4-aminopyridine) reduces separation to below 200 ms showing increased synchrony of activity across adjacent segments. Increased synchrony, characteristic of epilepsy, is similarly observed in genetic seizure mutants: bangsenseless^1^ (*bss*^*1*^) and paralytic^*K1270T*^ (*para*^*K1270T*^). Exposure of *bss*^*1*^ to clinically-used antiepileptic drugs (phenytoin or gabapentin) significantly reduces synchrony. In this study we use the measure of synchronicity to evaluate the effectiveness of known and novel anticonvulsive compounds (antipain, isethionate, etopiside rapamycin and dipyramidole) to reduce seizure-like CNS activity. We further show that such compounds also reduce the *Drosophila* voltage-gated persistent Na^+^ current (I_NaP_) in an identified motoneuron (aCC). Our combined assays provide a rapid and reliable method to screen unknown compounds for potential to function as anticonvulsants.

## Introduction

Epilepsy, which is the recurrence of spontaneous and seemingly unprovoked seizures, is a significant clinical disorder affecting ~1% of the global population. Treatment is commonly through chronic administration of antiepileptic drugs (AEDs), many of which can have severe side-effects. Drug treatment also has the severe limitation in that only about two-thirds of patients show reduction in frequency of seizure occurrence. For the remaining third, no effective treatment is currently available [1]. Genetics is believed to be a primary cause of many types of epilepsy and the number of genetic loci considered to be contributory to seizure exceeds 500 [2]. Of these loci, mutations in neuronal sodium channels are considered to be the most common genetic cause of epilepsy in humans [3]. Identification of genetic mutations offers the possibility to identify novel targets to facilitate next-generation AED design.

*Drosophila* has been used as a model organism to study seizure for over four decades [4, 5]. The comparably simple nervous system of both adult fly and larvae, coupled to genetic tractability, makes this insect attractive for studying the mechanisms that underlie seizure events. Molecular screens have also been used to identify seizure-suppressor genes, the identity of which can greatly facilitate not only better understanding of the seizure process but also identify possible targets for AED design [6–8]. However, molecular screens often generate lengthy lists of genes which require validation. This process can often serve as a bottleneck to progress. In this study we have capitalised on the relatively recent development of genetically encoded calcium indicators (termed GECIs) that allow neuronal activity to be imaged [9]. We exploit the fact that seizures are commonly associated with increased neuronal activity which often manifests as increased synchronicity in neuronal populations [10]. Here, we selectively express GCaMP5 in different sets of *Drosophila* motoneurons and measure synchrony of calcium activity between those neurons as a proxy for both seizure and, importantly, to determine potency of novel compounds identified to have anticonvulsive properties from behavioural screens.

The mammalian genome encodes nine voltage-gated sodium (Na_V_) alpha subunits, seven of which are expressed in neurons. By comparison, *Drosophila* expresses just a single, well-conserved (~50% amino acid identity [11]), homologue termed *paralytic* (*DmNa*_*V*_). As in humans, certain mutations of the *paralytic* gene result in a seizure phenotype (termed bang-sensitive). The name bang-sensitive derives from the heightened responses of mutant flies to mechanical shocking that induces a seizure-like behavior [12]. A similar phenotype can be induced in *Drosophila*, following exposure to proconvulsants picrotoxin (PTX) and 4-aminopyridine (4-AP). PTX is a Cl-channel inhibitor which blocks, amongst other channels, the GABA_A_ receptor whilst 4-AP blocks fast-activating K^+^ channels (in particular K_v_1). In this study, we use two DmNa_V_ mutations, in addition to these proconvulsants. The *bss*^*1*^ mutant carries a missense mutation in the paralytic voltage-gated sodium channel (Na_V_) and causes disruption in the function of the “paddle motif” which is implicated in the inactivation of the channel [13, 14]. This results in a gain-of-function. The *para*^*K1270T*^ (GEFS^+^) mutant models the febrile seizures associated with an SCNA1 mutation which was introduced at the comparable position into the *paralytic* gene [15].

We show that imaging calcium-induced activity with GCaMP5 expression in motoneurons correlates well with action potential firing in these neurons, as measured by simultaneous loose-patch recordings. Calcium imaging shows three types of activity waves reflective of forward, backward and turning locomotion. These activity waves, which pass between adjacent segments, are temporally well separated leading to a low measure of synchronicity in neuron activity between adjacent segments. Exposure to proconvulsants degrades the spatial separation of these waves and results in increased synchrony of activity between neurons in adjacent segments. This same increase in synchronicity is also observed in both *bss*^*1*^ and *para*^*K1270T*^ (GEFS+) mutants. Prior treatment of *bss*^*1*^ with AEDs, either phenytoin (Phy, which blocks I_NaP_) or gabapentin (Gbp, mixed action resulting in potentiation of GABA transmission), is sufficient to reduce synchronicity towards the wildtype level. We exploit the simple measure of synchronicity to show that novel compounds, identified from a previous behavioural screen, also reduce synchronicity which is consistent with having significant anticonvulsive properties in *Drosophila*.

## Materials and Methods

### Fly stocks

Flies were maintained on standard corn meal medium at 25°C. Temperature-sensitive stocks and their controls were maintained at 22°C. Wild-type flies were Canton-S. GCaMP5G; RRa and GCaMP5G; OK371-GAL4 stocks were generated by us through crossing of Bloomington stock 42037 (UAS-GCaMP5G) to RRa-Gal4 or OK371-Gal4 (vGlut, provided by Dr. James Hodge, University of Bristol). Bss^1^ (*para*^*L1699F*^) was originally provided by Dr. Kevin O’Dell (University of Glasgow) and is described in [14]. The *para*^*K1270T*^ GEFS^+^ line and the corresponding control line, *para*^*K1270K*^, containing a wild-type substitution, were provided by Dr. Diane O’Dowd (University of California, Irvine) and are described in [15]. RRa-GAL4 was crossed into these lines by standard genetics. The resultant stock was crossed to UAS-GCaMP5G and because the *para* gene is located on the X chromosome, only F1 male larvae were used (*para*/Y). RNAi transgene expression was achieved by crossing an RNAi line to *bss*^*1*^; RRa-Gal4::UAS-GFP^CD8^ and using male F1 larvae.

### Dissection, on-cell electrophysiology and calcium imaging

CNS was removed from wall climbing third instar larvae and placed dorsal surface up on a sylgard-coated coverslip (Dow Corning, MI, USA). Saline was composed of (in mM): 135 NaCl, 5 KCl, 4 MgCl_2_, 2 CaCl_2_, 5 TES, and 36 sucrose, pH 7.15 with NaOH. The ventral nerve cord (VNC) which contains motoneurons was viewed using an Olympus BX51-WI compound microscope with a 20x water-immersion lens. For electrophysiology, the neurolemma (glial sheath) covering the VNC was removed as described in [16] to gain access to the aCC motoneuron. Loose-patch recordings were obtained using a MultiClamp 700B amplifier and Digidata 1440A (Molecular Devices, Sunnyvale, CA). Borosilicate glass capillaries were used to pull recording electrodes (unpolished) with resistances between 3–5 MΩ. Data were acquired with a sampling rate of 20 kHz, filtered with a low-pass filter of 10 kHz and analysed in Clampfit 10.4 (Molecular Devices). Excitation of GCaMP was achieved using an OptoLED system (Cairn Research) with a 470 nm LED. The frame rate of acquisition was 5/s and frame duration of 200 ms with a QImaging Exi aqua camera (Photometrics, UK).

### Drug feeding

Phenytoin (sodium salt) was made as a stock solution (10 mg per ml) in 0.033 M NaOH. A 100 μl aliquot containing 0.4 mg/ml was added to liquid fly-food (5ml), which was then allowed to cool and set in a standard plastic fly vial. Gabapentin, antipain and isethionate were dissolved in water and added at concentrations of 0.2, 0.4 and 0.8 mg/ml, respectively. Etopiside was dissolved in ethanol:DMSO (5:1) and added at 0.8 mg/ml. Dipyramidole (0.8 mg/ml) and rapamycin (0.04 mg/ml) were dissolved in DMSO. Virgin flies and males (ratio ~ 2:1) were added to the food surface and allowed to lay eggs, the developing offspring were raised on this food until wall-climbing third instars emerged. Drug concentrations were derived from previous screens to identify optimal doses to rescue seizure behaviour in the *bss*^*1*^ mutation ([17] and unpublished). It is not meaningful to equate these concentrations to clinically-used drug dosages in humans given the uncertainties relating to how much is ingested by larvae and how much that is ingested is taken up into the CNS. All drugs were from Sigma (Poole, UK)

### Cross-correlation analysis

We used cross-correlation to analyse the extent to which motoneurons in adjacent segments showed synchronised activity. Imaging files were opened in Clampfit 10.4 (Molecular Devices) and analysed using the cross-correlation analysis tool with 16 lags (= 3200 ms). This tool allowed us to examine for time-shifts and cycles between imaging traces obtained from different cells of each preparation. The output of this analysis is plotted as correlation coefficient vs. lag time. The peak of this curve is the time shift that leads to the best overlap of the compared traces. We considered a peak centred around 0 ms (range ± 199 ms) as synchronised. Peaks that lay outside of this range (≥ ± 200 ms) were considered asynchronous.

### Whole-cell Electrophysiology

Third instar CNS was dissected and fixed to a sylgard-coated coverslip (Dow Corning, MI, USA) with cyanoacrylate glue (Vetbond, WPI, Stevenage, UK). Rupture of the neurolemma surrounding the CNS was as described [16] but with the larger third instar CNS requiring a slightly larger bore micropipette. Whole-cell voltage clamp recordings were made using thick-walled borosilicate glass electrodes (GC100F-10; Harvard Apparatus, Edenbridge, UK), fire polished to 10–15 MΩ. aCC motoneurons were identified by GFP expression. Recordings were made using an Axon 1D amplifier controlled by pClamp (v10.2, Molecular Devices, Sunnyvale, CA). Recordings were sampled at 20kHz and filtered online at 10kHz. Traces were leak-subtracted using a standard P/4 protocol. This protocol use prepulses (repeated 4 times) at one quarter strength of the test pulse to enable leak-currents (those due to passive characteristics of the membrane) to be subtracted from the test pulse to isolate only the active (I_Na_) current. Capacitance was measured by integrating the area under the capacity transient resulting from a step protocol from -60mV to -90mV. Only cells with R_in_ ≥ 500 MΩ were accepted for analysis. The stimulation protocol was run 4 times for each cell and a composite average used for analysis. Because we observe a change to I_NaP_ and not I_NaT_, we calculated the ratio of these two conductances, which are mediated by the same ion channel. Peak I_NaT_ was measured using the cursor function in Clampfit whilst I_NaP_ was measured as the average current between two cursors spanning a 100 ms duration of the current step (340–440 ms). These two current values were then used to calculate the ratio.

Sodium conductance saline consisted of (in mM): 100 NaCl, 6 KCl, 50 TEA, 10 4-aminopyridine, 10 MgCl_2_, 10 HEPES, and 10 sucrose, pH 7.4. Internal patch saline consisted of (in mM): 140 CsCH_3_SO_3_, 5 CsCl, 2 MgCl_2_, 11 EGTA, and 20 HEPES, pH 7.4.

## Statistics

Significance of the differences in level of synchrony was tested using an un-paired Student’s t-test and were comparisons between a single test and a single control mean value. Cross-correlation analyses were tested for significance using the Fisher’s exact test. Analysis of I_NaP_/I_NaT_ values was performed by one-way ANOVA using the Bonferroni post-hoc test. Results were deemed significant at P ≤ 0.05 (*), P ≤ 0.01 (**) or P ≤ 0.001 (***).

## Results

### Combined calcium imaging and loose-patch recordings

**Fig 1:**
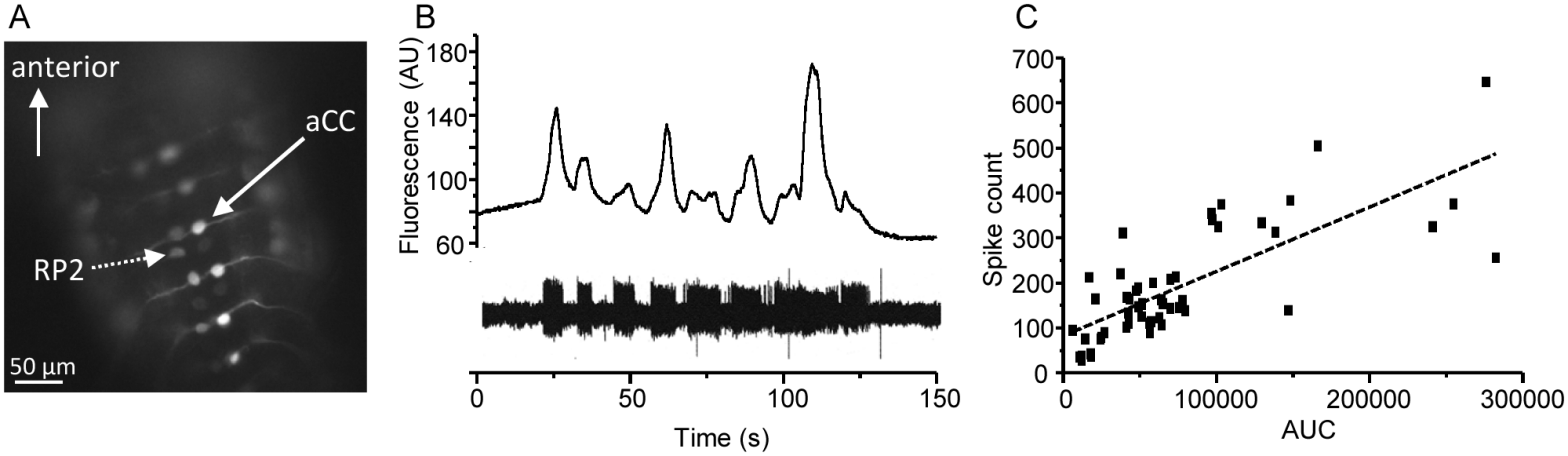
GCaMP imaging reports neuronal activity. A) Expression pattern of GCamP5 using the RRa-Gal4 driver line. Paired aCC neurons (solid arrow) show clear segmental expression whilst RP2 neurons (dashed arrow) show weaker expression and only in some segments. B) Sample recording of simultaneous loose-patch (lower trace) and calcium-imaging (upper trace) of the same aCC motoneuron. C) Spike count from loose-patch recordings of aCC neurons plotted against the area under the curve (AUC) of calcium-imaging from the same motoneuron (accumulated data from n = 5). The line of fit shown has an R^2^ = 0.572.

The use of the calcium reporter GCaMP for reporting neuronal activity has grown considerably over recent years [18]. However, in the large majority of these studies validation of a linear relationship between GCaMP-signal and action potential firing has not been established. Thus, prior to using this technique to image activity, we verified that the calcium signal we obtain from GCaMP expression in motoneurons of third instar isolated CNS mirror their electrical activity. We expressed GCaMP5 in aCC neurons using the RRa-GAL4 driver line. GCaMP5 is clearly expressed bilaterally in aCC neurons of all segments and also expressed in some, but not all, RP2 neurons (Fig 1A). We performed simultaneous on-cell (loose-patch) recordings from aCC neurons to allow recording of action potentials (Fig 1B, lower trace) and calcium-imaging (Fig 1B, upper trace). Peaks in GCaMP-induced fluorescence clearly coincided with bouts of action potentials. Moreover, plotting number of action potentials recorded vs. area under the curve (AUC) obtained from imaging shows a strong positive correlation between the two (R^2^ = 0.572, n = 51, P < 0.001, ANOVA) (Fig 1C). Thus, at least in motoneurons, GCaMP-induced fluorescence is a good indicator of neuronal activity. However, GCaMP imaging is not sufficient to resolve single action potentials and the correlation between GCaMP signal and loose-patch recordings is not perfect (which would equate to an R^2^ = 1.0). Future studies using genetically-encoded voltage reporters may overcome these issues.

### Movement patterns acquired from the isolated VNC

**Fig 2:**
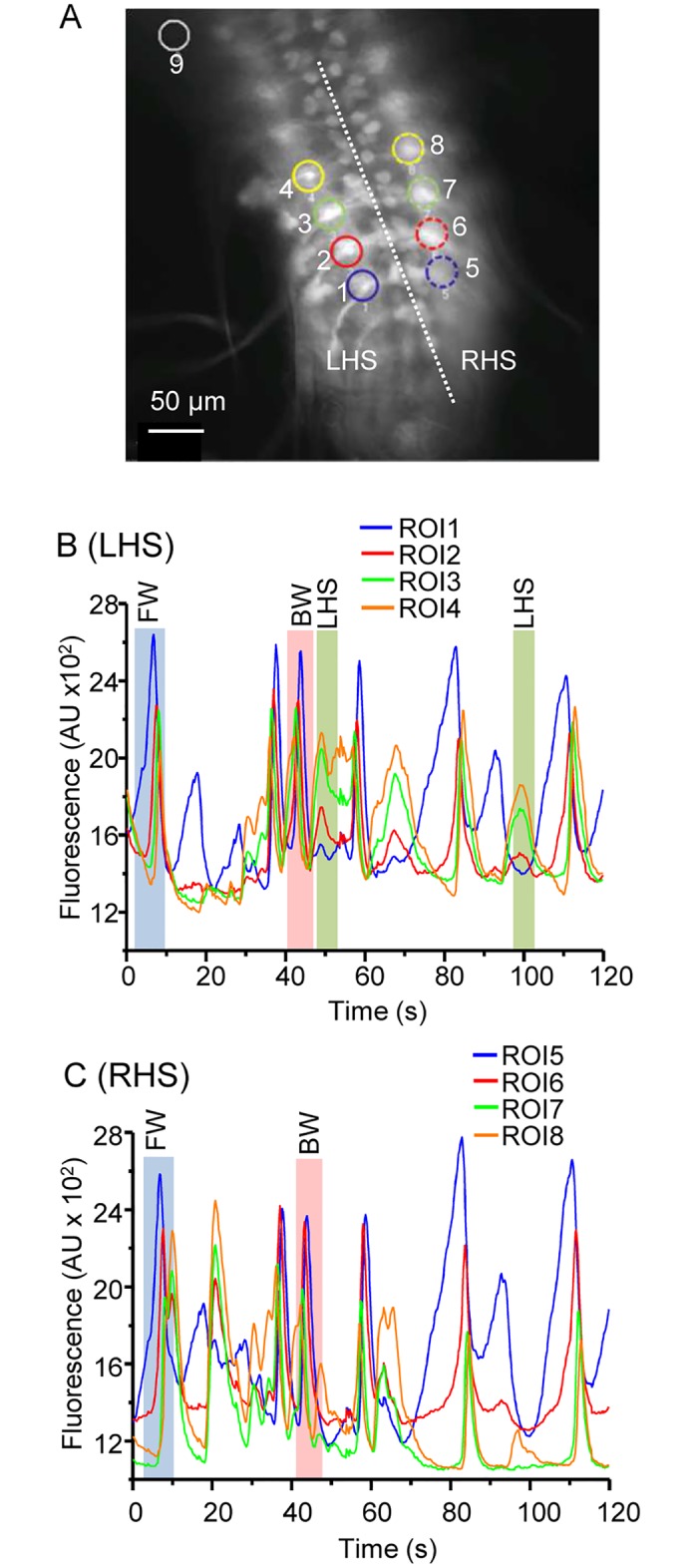
Isolated VNCs show patterned GCaMP activity. A) Expression pattern of GCaMP in the OK371-Gal4 (vGlut) driver line. Regions where motoneuron dendrites overlap were chosen as regions of interest (ROI) to monitor segmental calcium levels over time. A ROI was also drawn outside of the VNC for background normalization (upper left corner). The *Drosophila* VNC is symmetrical across the midline (dotted white line). L/R HS = left and right hand side, respectively. B, C) Activity patterns observed from the left and right hand side of the same VNC. Waves starting at the posterior end of the VNC (FW, forward locomotion) and waves starting at the anterior end of the VNC (BW, backward locomotion) are evident in both sides whilst unilateral activity (turning, only visible in panel B) is only seen one side (labelled LHS).

Our overall goal was to determine how neuronal activity is affected in the CNS of defined seizure mutants. Thus, we first established the activity patterns of motoneurons during wildtype larval fictive movement. We expressed GCaMP5 with the OK371-Gal4 line, which drives nearly exclusively in motoneurons (Fig 2A) [19]. We imaged the isolated CNS of third instar larvae and constructed regions of interest (ROIs) around the dendritic areas of single adjacent segments. These areas represent convergence points for the axons of multiple motoneurons. We observed three consistent patterns of activity: (i) activity starting from the posterior of the VNC moving toward the anterior (representing forward locomotion). (ii) Anterior to posterior waves, representing backward locomotion and (iii) activity exclusively on one side or the other of the VNC (representing bending or turning behaviour) (Fig 2B and 2C). Our findings are in good agreement with a recent study that details the imaging of fictive movement in *Drosophila* larval VNC in greater detail [20]. The frame rate we used (5Hz) was chosen as a compromise between speed and file size. In the isolated CNS a full forward peristaltic wave takes ~ 10 s to traverse the larval body, which equates to ~1 s per segment [20]. Thus, 5Hz is sufficient to capture this transition but future studies would be improved by increasing frame rate up to a desired rate of 20Hz (every 50 ms).

### Exposure to proconvulsants increases coincidence of neuronal activity

**Fig 3:**
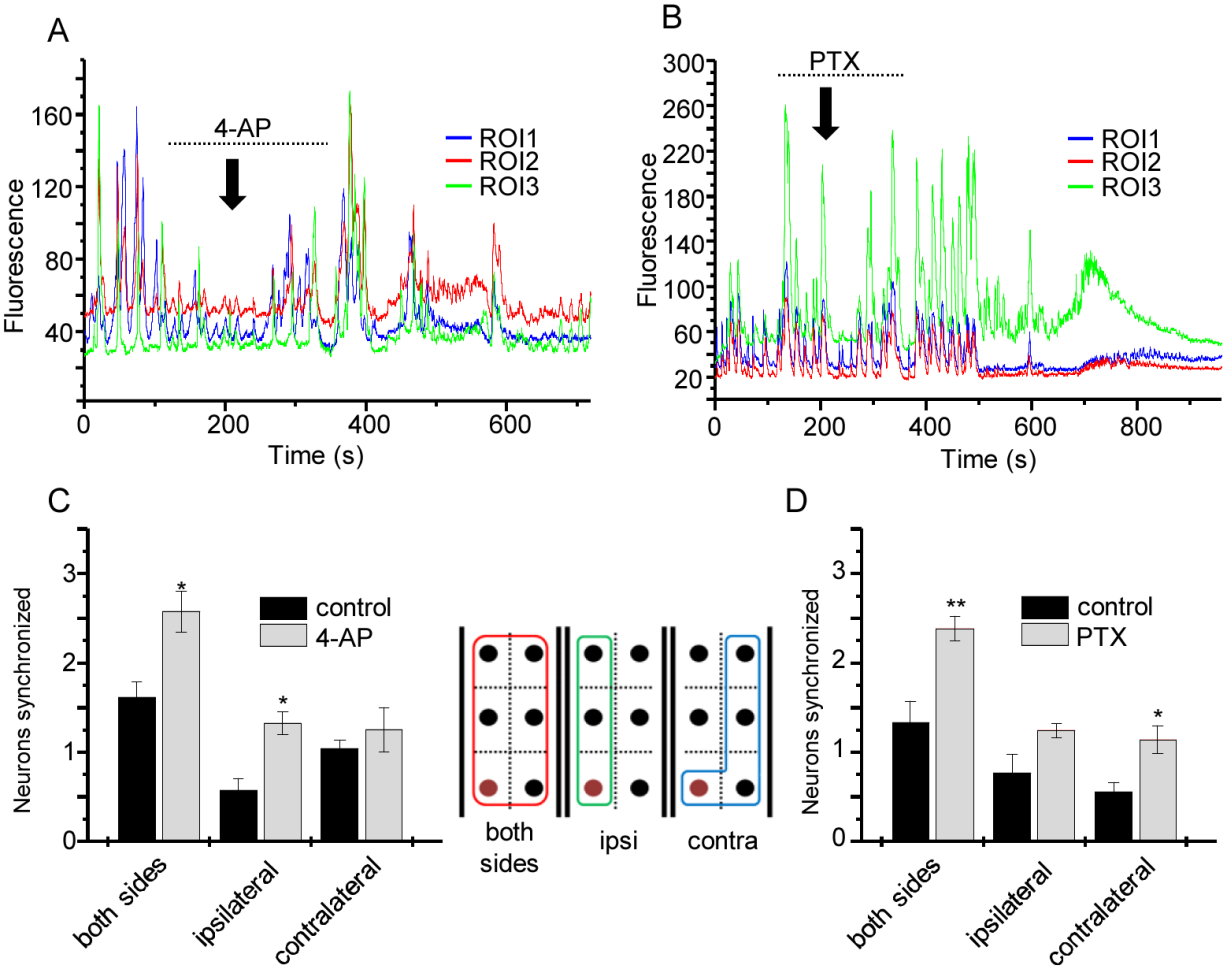
Proconvulsants increase synchronicity of neuron activity. A) GCaMP5 fluorescence from single aCC neurons from consecutive segments show hyperactive and synchronised pattern changes following 4 min application of 4-AP (3 mM). Dotted line shows period when 4-AP was perfused and downward arrow indicates time 4-AP entered the bath. The time difference results from the length of tubing between gravity-driven perfusion system and the recording chamber. B) PTX (5μM) perfusion similarly increases neuron activity. Dotted line shows period when PTX was perfused and downward arrow indicates time PTX entered the bath. C, D) Analysis of number of synchronised aCC cells before and after 4-AP (C) and PTX (D) application (values are mean ± sem for n = 6 and 5, respectively). Synchrony was compared between a fixed ROI (labelled brown in the inset) and compared to all ROIs (red box on inset) and with those on the ipsilateral (green box) or contralateral (blue box) sides.

A characteristic of epileptiform activity is increased synchrony of action potential firing between neurons [10]. To determine whether an increase in synchrony can be observed in the *Drosophila* CNS, we expressed GCaMP5 in aCC motoneurons and perfused the isolated preparation with saline containing either the proconvulsant compound 4-AP (3 mM, Fig 3A) or PTX (5 μM, Fig 3B) for a period of four minutes. To measure synchronicity, ROIs were drawn around single aCC neurons from three consecutive segments. ROI 1 was used as a reference trace and the peak coincidence of other ROI traces was analysed by comparison. Application of 4-AP induced a significant increase in synchronicity. This effect was strongest when all ROIs on both sides of the VNC were compared to ROI 1 (termed ‘both sides’ on Fig 3), but was also observed between ipsilateral ROIs in adjacent segments (Fig 3C). By contrast, no significant change was observed between synchronicity of contralateral ROIs neurons compared to ROI 1. PTX exposure produced a similar and significant increase in synchronicity when all or the contralateral ROIs were compared to ROI 1. The synchronicity observed between ipsilateral ROIs across segments was not significantly different (Fig 3D). Thus, we conclude that exposure to proconvulsants significantly increases synchronicity of neuron activity between adjacent segments that normally show a clear demarcation of activity in wildtype.

### Cross-correlation analysis identifies synchronised activity in motoneurons in seizure mutants

**Fig 4:**
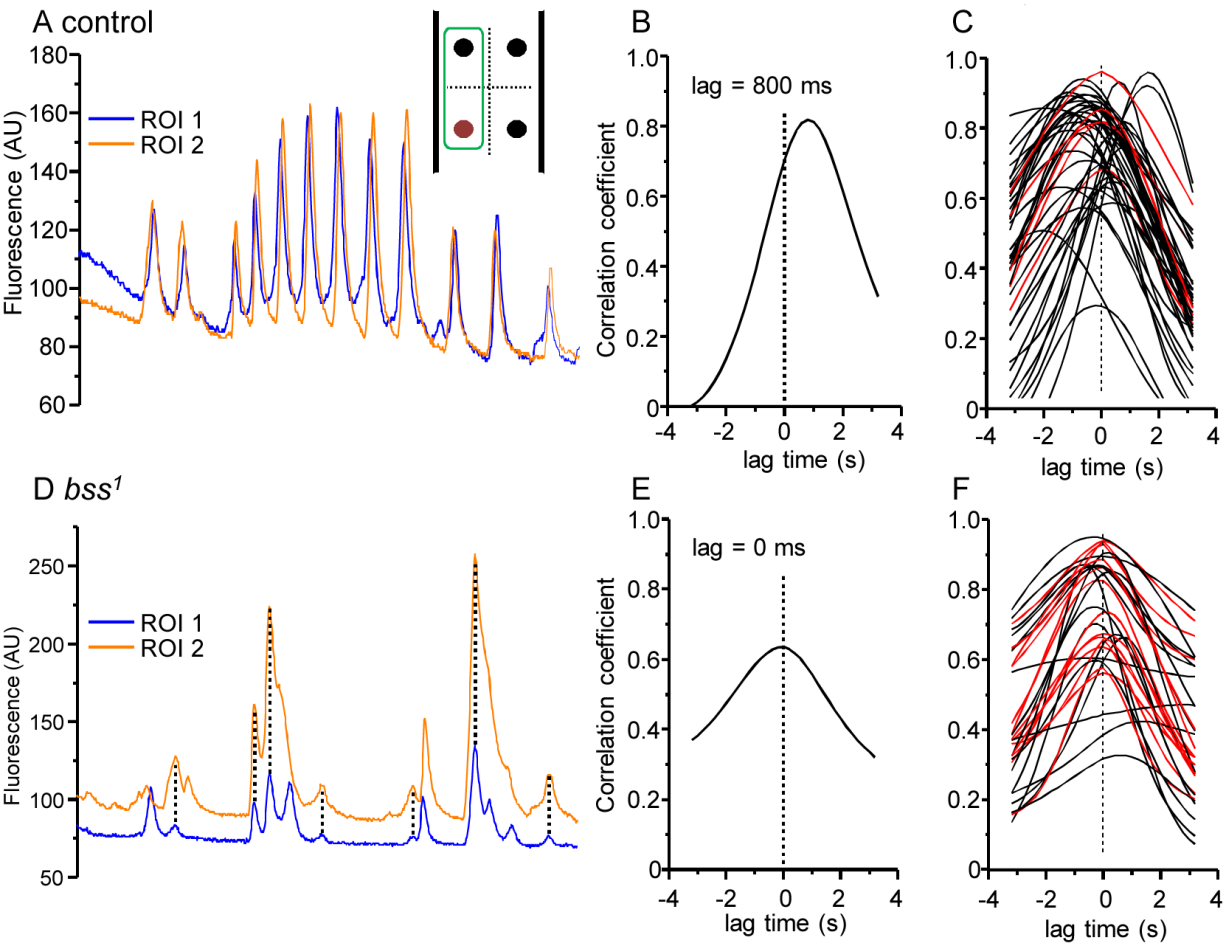
bss^1^ mutants show increased synchronicity of neuronal activity. A) Representative calcium-imaging traces from wildtype aCC neurons in two adjacent segments (i.e. ipsilateral, see inset). B) Results of cross-correlation for the traces from the recording shown in A). The peak of the curve (at 800 ms) shows the lag time which presents the best alignment of both traces. C) Peaks plotted for recording from multiple preparations show the majority (black) peak outside of the region considered to show synchronicity. Those that are considered synchronous (i.e. occur within ≤ 199 ms) are shown in red. D) A calcium-imaging trace from two adjacent aCC neurons in the *bss*^*1*^ background. Coincident peaks are marked with dashed lines. E) Cross-correlation for the traces from the recording shown in D). The peak of the curve (at 0 ms) is indicative of synchronised activity. F) Peaks plotted from multiple *bss*^*1*^ preparations. Synchronised peaks are shown in red and asynchronous in black.

The proconvulsive compounds, 4-AP and PTX, significantly increase synchrony of activity between neurons in wildtype backgrounds. Such activity is characteristic of seizures and because of this we asked whether characterised *Drosophila* seizure mutants similarly showed increased synchronicity. We imaged two such mutants: *bss*^*1*^ and a knock-in model of GEFS+ (*para*^*K1270T*^). Both mutants show seizure-like activity in response to either a mechanical or electric shock (*bss*^*1*^) or elevated temperature (*para*^*K1270T*^). Each image recording was analysed using a standardised and more rigorous procedure that involved a cross-correlation analysis between individual aCC neurons of adjacent segments (see materials and methods). A typical recording in wildtype shows relatively few simultaneous peaks of calcium-activity in ipsilateral aCC neurons between adjacent segments (Fig 4A) and the resulting curve from cross-correlation analysis shows that the lag time between the two ROIs is ~800 ms (Fig 4B). Overlaying cross-correlation curves from multiple wildtype recordings reveals that the large majority exhibit positive or negative lag time values greater than 200 ms (Fig 4C, curves shown in black) while only a minority (11.6%) are synchronised indicated by a lag time of ≤ 199 ms (Fig 4C, curves shown in red). By contrast, identical recordings of GCaMP activity in *bss*^*1*^ show 42.0% synchronised activity (Fig 4D–4F). Analysis of statistical significance using a Fisher’s exact test shows this difference is significant at P ≤ 0.001 (Table 1).

**Table 1:** Summary of results showing degree of synchrony on aCC activity imaged using GCaMP.

Genotype	n	synchrony	asynchrony	% synchrony
wildtype	43	5	38	11.6
*bss*^*1*^	50	21	29	42.0***
GEFS+ control (K1270K) (21°C)	32	6	26	18.8
GEFS+ (K1270T) (21°C)	31	12	19	38.7*
GEFS+ control (K1270K) (28°C)	23	2	21	8.7
GEFS+ (K1270T) (28°C)	22	9	13	40.9**
*bss*^*1*^ + NaOH	27	12	15	44.4
*bss*^*1*^ + Phy (0.4 mg/ml)	37	9	28	24.3*
*bss*^*1*^ + water	17	11	6	64.7
*bss*^*1*^ + Gbp (0.2 mg/ml)	14	3	11	21.4*
*bss*^*1*^ + antipain (0.4 mg/ml)	30	9	21	30.0*
*bss*^*1*^ + isethionate (0.8 mg/ml)	30	10	20	33.3*
*bss*^*1*^ + EtOH/DMSO (5:1)	19	12	7	63.2
*bss*^*1*^ + etopiside (0.8 mg/ml)	31	9	22	29.0*
*bss*^*1*^ + DMSO	20	12	8	60
*bss*^*1*^ + dipyramidole (0.8 mg/ml)	33	6	27	18.2**
*bss*^*1*^ + rapamycin (0.04 mg/ml)	27	8	19	29.6*

Phy = phenytoin. Gbp = gabapentin. Compounds are grouped according to solvent used.

P ≤ 0.05 (*),

P ≤ 0.01 (**) or

P ≤ 0.001 (***).

Next we expressed GCaMP in aCC in the *para*^*K1270T*^ background and the corresponding control line *para*^*K1270K*^. Analysis of synchronicity at room temperature (~21°C) shows a similarly heightened level of synchronicity of activity (38.7%) compared to wildtype controls (18.8%, P = 0.05, Table 1). This is similar to the change in synchronicity observed in *bss*^*1*^ indicative that activity patterns are altered even in the ‘non-seizure’ state. The *para*^*K1270T*^ mutation can be induced to show a seizure phenotype when elevated to 28°C [15]. Thus we repeated our analysis at this higher temperature. Synchronicity increased to 40.9% whilst wildtype controls showed slightly reduced synchronicity at this temperature (8.7%, Table 1). Thus, two independent mutants, that both result in seizure-like activity, show increased synchronicity of neuronal activity, mirroring the effects seen with proconvulsants.

### Antiepileptic drugs reduce synchronicity

It is well established that feeding the antiepileptic drugs Phy and Gbp to *Drosophila* is sufficient to reduce seizure duration in many genetic seizure mutants [21, 22]. Thus, we asked whether these drugs, as we might predict, reduce synchrony in *bss*^*1*^ by raising larvae on food containing either Phy (0.4 mg/ml) or Gbp (0.2 mg/ml). After exposure to Phy, synchronicity in *bss*^*1*^ was reduced (44.4 to 24.3%, P = 0.05, Table 1). Exposure to Gbp also decreased synchrony (64.7 to 21.4%, P = 0.02, Table 1). These results strongly support the hypothesis that increased synchrony of neuron activity, seen in both proconvulsant-treated and genetic seizure mutants, contributes to the seizure phenotype.

### Determination of synchrony of activity as a diagnostic tool for identification of AEDs

Measurement of neuronal activity using GCaMP is relatively straight-forward and, moreover, could be scaled up to support medium to high-throughput drug screening. In a previous study, we used an RNAi-based screen to identify genes which, on knock-down, are anti-convulsive in *Drosophila* [17]. We found ~90 such genes including *raptor*, *phosphodiesterase 11*, *topoisomerase II*, *cyclin-dependent kinase 4* and a *serine-type peptidase*. Based on known gene function, we identified chemical inhibitors and showed that these were similarly anticonvulsive when fed to *bss*^*1*^ seizure mutants. These compounds were rapamycin (*raptor*), dipyramidole (*phosphodiesterase 11*), etopiside (*topoisomerase II*), isethionate (*cyclin*-*dependent kinase 4*), and antipain (*serine*-*type peptidase*). Feeding of these compounds to *bss*^*1*^ larvae also significantly reduced synchronicity of activity (see Table 1). This observation not only confirms the potential for these compounds to act as templates to identify novel AEDs, but also shows the tractability of this technique to act as a secondary screen to validate compounds identified from behavioural testing.

### Novel compounds target I_NaP_

The compounds, antipain, isethionate, etopiside, dipyramidole and rapamycin were identified from a prior RNAi-screen for splicing regulators of *DmNa*_*v*_ [17]. This screen identified genes which, on knock-down, promoted version-K of exon 25 to be spliced in at the expense of the mutually-exclusive exon L. Inclusion of exon K results in a channel that supports a significantly smaller voltage-gated persistent Na^+^ current (I_NaP_) [23]. Reduction of I_NaP_ is anti-convulsive in both *Drosophila* and humans, the latter based on the observations that I_NaP_ is often increased by seizure-inducing mutations in Na_v_s and that AEDs such as Phy specifically target this current component [22, 24, 25]. Thus, we hypothesised that the effects of these novel compounds would include reduction of I_NaP_. We have already shown this to be the case for dipyramidole [17].

**Fig 5:**
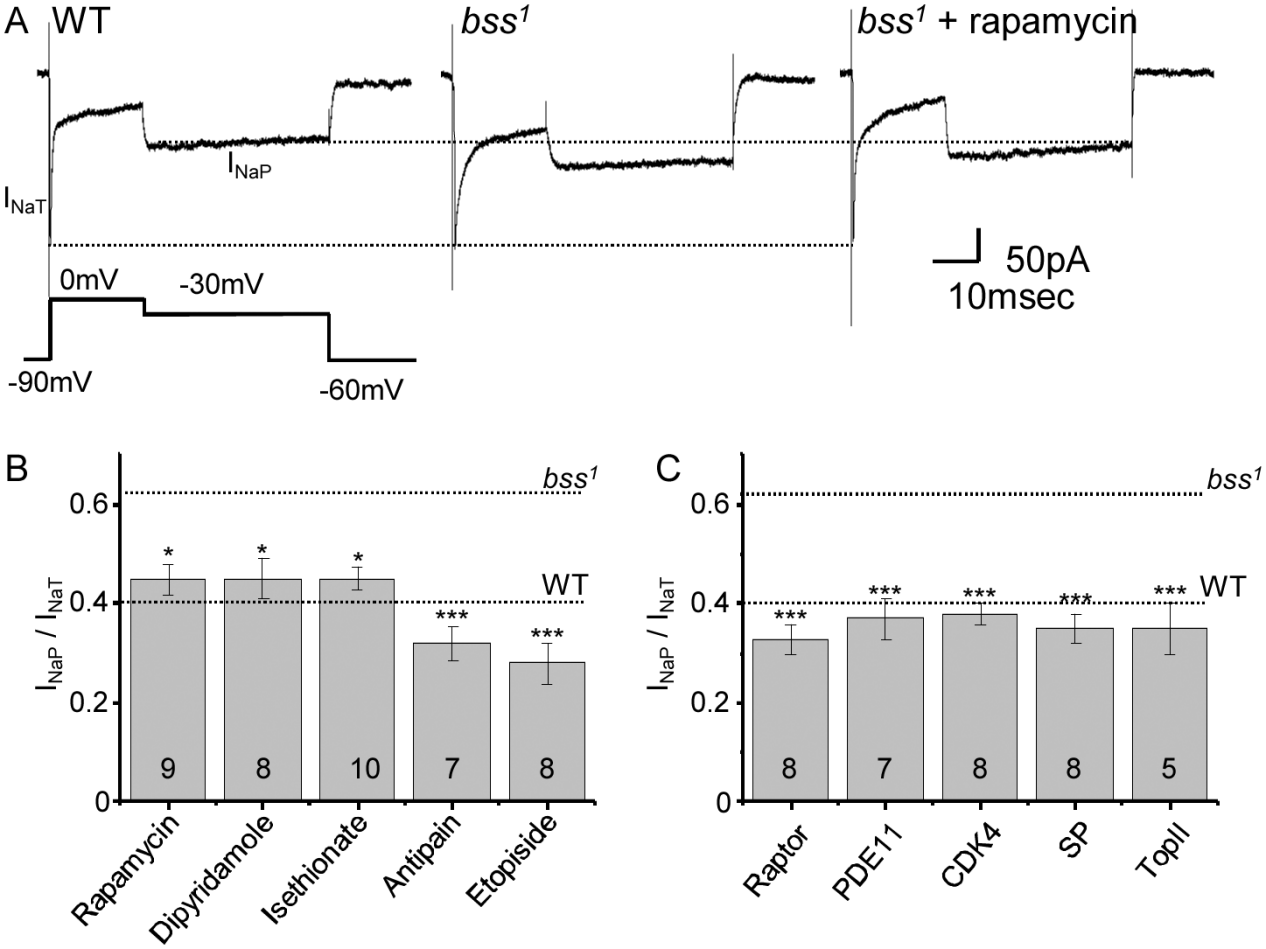
anticonvulsive compounds reduce I_NaP._ A) I_NaP_ is increased in *bss*^*1*^ relative to wildtype (WT). I_NaP_ was evoked using a voltage protocol that maintained the aCC neuronal membrane at 0mV to inactivate I_NaT_ before dropping to -30mV to measure maximal I_NaP_. Treatment with rapamycin reduces I_NaP_ back to WT level. The three recordings shown were chosen because they have the same amplitude of I_NaT_. The dotted lines are drawn as indictors only to show amplitudes of I_NaT_ and I_NaP_ in WT. B) Ratio for I_NaP/NaT_ recorded in aCC for *bss*^*1*^ raised in food containing either rapamycin, dipyramidole, isethionate, antipain or etopiside (concentrations as per Table 1). Values for untreated *bss*^*1*^ and WT are shown for reference. C) Ratio for I_NaP/NaT_ for *bss*^*1*^ following expression of RNAi transgenes targeting *raptor*, *phosphodiesterase 11* (PDE11), *CDK4*, *serine*-*type peptidase* (SP) or *topoisomerase II* (TopII). Values are means ± sem.

To test this, we measured I_NaP_ in aCC motoneurons using whole cell voltage clamp. I_NaP_ is greatly increased in *bss*^*1*^ which undoubtedly contributes to the seizure phenotype of this mutant [17]. The fast component, I_NaT_, is not affected resulting in an increase in the I_NaP/NaT_ ratio (Fig 5A and see [17]). Exposure to all five novel compounds (at the concentrations stated in Table 1) reduced I_NaP_ to restore the I_NaP/NaT_ ratio back to wildtype levels (Fig 5B). We also tested gene knock-down, using neuronal expression of RNAi transgenes targeted at the original genes identified in our screen (the protein products of which are inhibited by the chemical compounds). RNAi expression was restricted to aCC in the *bss*^*1*^ background, which also contained a GFP transgene to label aCC neurons. Knockdown of *CDK4* (isethionate), *serine-type peptidase* (antipain), *topoisomerase II* (etopiside), *phosphodiesterase 11* (dipyramidole) or *raptor* (rapamycin) similarly reduced I_NaP_ to rescue the I_NaP/NaT_ ratio back to wildtype level (Fig 5C). This effect validates our original screen and provides significant data to warrant further investigation of the utility of using these chemical inhibitors to prevent seizure in mammals.

## Discussion

Behavioural screens in animals such as *Drosophila* or zebrafish offer the prospect to undertake high-throughput screens to identify novel hit compounds for the development of more efficacious drugs to treat human disease. The use of an *in vivo* screen diminishes the requirement for pre-determined targets but, on the other hand, necessitates an appropriate post-screen validation step. This step is essential to deselect compounds that although produce the same change in behaviour, do so through undesirable mechanisms. Maintaining capacity for high-throughput post-screen validation is often challenging. In this study we show that calcium imaging may provide medium-throughput validation for behavioural screens performed in *Drosophila* to identify potential anticonvulsive compounds [17]. A similar approach has been proposed in zebrafish, but has not yet been fully developed [26]. The ability to image single motoneurons, the activity of which reflects the larger motoneuron pool, provides not only excellent signal-to-noise, but also enables accurate measurement of activity synchronicity between adjacent neurons. Given the relative simplicity of imaging involved, this preparation should lend itself to being scaled-up. This endeavour may be facilitated by imaging directly through the larval cuticle which will undoubtedly prolong preparation lifetime and negate the need for CNS removal rendering the technique non-invasive [27].

Calcium imaging using genetically-encoded reporters serves as a proxy for recording action potentials in neurons and is far less damaging than either traditional small molecule dyes or microelectrodes. The GCaMP family of reporters, in particular, have attracted much attention since their development and their use is now widespread in many animal models [28]. It is important that their use is ‘calibrated’ prior to use to establish that they are able to report over a linear range of activity of the imaged neurons. We show that for the aCC motoneuron this is indeed the case. Our choice to use this motoneuron is guided by the ability to combine genetics and electrophysiology: selective GAL4 drivers exist to express UAS-transgenes in this neuron which is also accessible to patch electrodes. That I_NaP_ is greater in amplitude in aCC motoneurons in seizure mutants is indicative that they share properties with central interneurons in human epilepsy which can also show increased I_NaP_ [25].

The utility of imaging of neuronal activity is based on the observation that epilepsy is a disorder often associated with excessive synchronization of large neuronal populations [29]. That we show synchronicity of neural activity increases between motoneurons in *Drosophila* CNS, following exposure to known proconvulsants, underscores the utility of this insect model for seizure-related research. Indeed, seizures in both humans and *Drosophila* exhibit sufficient parallels to implicate that the underlying neuronal abnormalities are highly similar. Previous investigations have shown in *Drosophila* that seizures include 1) a defined seizure threshold, 2) genetic mutations that modify seizure-susceptibility, 3) electroconvulsive shock therapy raises the threshold for subsequent seizures, 4) seizures spread throughout the CNS along defined neuronal tracts, 5) seizures can be localised to distinct regions of the CNS and 6) seizures can be ameliorated by anti-epileptic drugs used to treat human epilepsy [21, 30–32]. The availability of single-gene mutants (either spontaneous or knock-in) that both reduce seizure threshold and increase seizure duration provide an ideal platform for undertaking high-throughput screens. Our demonstration that neuronal synchrony in such genetic backgrounds mirrors that observed in wildtype exposed to proconvulsant also serves to support using the latter to study seizure mechanisms and identify novel anticonvulsant compounds.

In a previous high-throughput RNAi screen, which utilised luciferase-based reporters of DmNa_v_ splicing, we identified 90 genes that on knock-down resulted in significant rescue of seizure duration in *bss*^*1*^. These genes included *CDK4*, *topoisomerase II*, a *serine*-*type peptidase*, *raptor* and *phosphodiesterase 11* [17]. Inhibitors for the respective protein products of these genes (isethionate, etopiside, antipain, rapamycin and dipyramidole) all rescue seizure in *Drosophila* and, as we show here, significantly reduce neuronal synchronicity in *bss*^*1*^. The Cyclin/CDKs have been implicated in epileptogenesis. This includes up-regulation of Cyclin B1 in hippocampus of pentylenetetrazole (PTZ)-kindled rats (Pavlova et al., 2006) and in human patients with temporal lobe epilepsy (Nagy and Esiri, 1998). The related Topoisomerase I has also been identified as a seizure-suppressor in *Drosophila* (Song et al., 2007). mTOR signalling is elevated following seizure in rodent models (Waltereit et al., 2006; Grabenstatter et al., 2014) and, moreover, rapamycin has potential anticonvulsive properties in the WAG/Rij rat absence seizure model (Russo et al., 2013) and in kianic acid-induced *status epilepticus* in rat (Macias et al., 2013). The role of phosphodiesterase inhibitors as anticonvulsants is more controversial. Inhibiting phosphodiesterase-5 with sildenafil is anti-convulsant in the mouse 6-Hz psychomotor seizure model [33] but exhibits pro-convulsant activity in a PTZ-induced mouse clonic seizure model [34]. Our ability to screen compounds for an ability to reduce synchronicity of neuronal activity shows that their effect is in keeping with that observed with clinically-used AEDs. Disruption of synchronicity of neuronal activity is generally believed to be anti-convulsive and in this regard these novel compounds may have potential to catalyse the development of novel AEDs [29]. However, the precise mode-of action of these compounds, with respect to reducing seizure, remains unknown and must await future studies in order to determine this.

In summary, we report the development of a technically-simple assay to measure synchronicity of activity in a well-defined locomotor circuit in *Drosophila*. The usefulness of imaging activity in such circuits extends beyond the identification of potential compounds as anticonvulsants. Nevertheless, we show here that such imaging provides excellent validation of mode-of-action for compounds identified through high-throughput screening for novel targets and compounds to catalyse the development of next-generation AEDs.
